# Articulating Video Stylet Compared to Other Techniques for Endotracheal Intubation in Normal Airways: A Simulation Study in Consultants with No Prior Experience

**DOI:** 10.3390/jcm13030728

**Published:** 2024-01-26

**Authors:** Simone Messina, Federica Merola, Cristina Santonocito, Marco Sanfilippo, Giulia Sanfilippo, Federica Lombardo, Andrea Bruni, Eugenio Garofalo, Paolo Murabito, Filippo Sanfilippo

**Affiliations:** 1 Department of Anaesthesia and Intensive Care, Policlinico-San Marco University Hospital, Via S. Sofia n 78, 95123 Catania, Italy; messina.simone05@gmail.com (S.M.); merolafede@gmail.com (F.M.); cristina.santonocito@gmail.com (C.S.); lombardofederica92@gmail.com (F.L.); paolomurabito@gmail.com (P.M.); 2 School of Anesthesia and Intensive Care, University “Magna Graecia”, 88100 Catanzaro, Italy; marco.sanfilippo.96@gmail.com (M.S.); giulia.snf@gmail.com (G.S.); andreabruni87@gmail.com (A.B.); eugenio.garofalo@gmail.com (E.G.); 3 Section of Anesthesia, Department of General Surgery and Medical-Surgical Specialties, University of Catania, 95124 Catania, Italy

**Keywords:** endotracheal intubation, manikin, video laryngoscopy, fiberoptic bronchoscopy, direct laryngoscopy

## Abstract

Simulation for airway management allows for acquaintance with new devices and techniques. Endotracheal intubation (ETI), most commonly performed with direct laryngoscopy (DL) or video laryngoscopy (VLS), can be achieved also with combined laryngo-bronchoscopy intubation (CLBI). Finally, an articulating video stylet (ProVu) has been recently introduced. A single-center observational cross-sectional study was performed in a normal simulated airway scenario comparing DL, VLS-Glidescope, VLS-McGrath, CLBI and ProVu regarding the success rate (SR) and corrected time-to-intubation (cTTI, which accounts for the SR). Up to three attempts/device were allowed (maximum of 60 s each). Forty-two consultants with no experience with ProVu participated (15 ± 9 years after training completion). The DL was significantly faster (cTTI) than all other devices (*p* = 0.033 vs. VLSs, and *p* < 0.001 for CLBI and Provu), no differences were seen between the two VLSs (*p* = 0.775), and the VLSs were faster than CLBI and ProVu. Provu had a faster cTTI than CLBI (*p* = 0.004). The DL and VLSs showed similar SRs, and all the laryngoscopes had a higher SR than CLBI and ProVu at the first attempt. However, by the third attempt, the SR was not different between the DL/VLSs and ProVu (*p* = 0.241/*p* = 0.616); ProVu was superior to CLBI (*p* = 0.038). In consultants with no prior experience, ProVu shows encouraging results compared to DL/VLSs under simulated normal airway circumstances and further studies are warranted.

## 1. Introduction

Performing endotracheal intubation (ETI) remains a crucial skill for the management of airways, and it is performed both under elective cases, as in the operating room during the induction of general anesthesia, and in more complex situations, for instance, in rapidly deteriorating hospitalized patients, as well as in the out-of-hospital setting. Hence, both anesthesiologists and other healthcare professionals (i.e., emergency physicians and paramedics) dealing with airway management require regular training to develop ETI skills [1,2].

In this regard, the use of simulation provides great opportunities to improve confidence and familiarity with airway management techniques (ETI, but also bag-mask ventilation and the placement of supraglottic airway devices), especially under difficult situations and for novices [3,4]; moreover, simulation offers the chance to gain experience with new equipment and devices [5]. Recently, in the context of the pandemic, simulation has also allowed for familiarization with procedures performed under the constraints of wearing personal protective equipment [6].

The direct laryngoscopy (DL) is the most widely used method to perform ETI; notably, it might a challenging learning curve [7,8], and the proficiency with DL deteriorates over time if not routinely practiced [9]. Alternatively, video laryngoscopes (VLSs) have improved the visualization of the vocal cords [10], with improved results suggested both in any setting [11] as well as in challenging scenarios such as cardiac arrest [12] or nasotracheal intubation [13]. Moreover, the VLS technique has recently shown better performance than DL in a large multicentric randomized controlled trial conducted mostly in the emergency department setting [14]. Hence, it is possible that VLS will gradually replace the use of DL not only in difficult scenarios but as a routine device for the approach to airway management.

However, even if glottis visualization with VLS is usually superior compared to DL [15,16], the VLS performance is influenced by the operator’s expertise and the device’s characteristics, even if the learning curve seems shorter than with DL [17,18]. In particular, the main challenge with VLSs is to properly direct the endotracheal tube (ETT) through the vocal cords, which has also been called the “laryngoscopy paradox”. In order to facilitate the task of properly guiding the ETT, it has been suggested to combine the use of VLS or DL (to open the upper airways) with the fiberoptic bronchoscope (FOB), in the so-called combined laryngo-bronchoscope intubation (CLBI) approach, which has been used in challenging clinical cases [19,20,21,22], even in the pediatric population [23]; moreover, the combined technique has been reported also with the use of FOBs with supraglottic airway devices [24,25]. Some preliminary studies on the CLBI approach have been conducted [26,27,28,29], but the results of these studies are probably significantly influenced by the operator’s skills in terms of the manipulation of the FOB. Alternatively, flexible (also called articulating) video stylets have been developed as an alternative option for video-assisted ETI. The shape of the newer video stylets as well as their handling and manipulation seem closer to the DL or VLS compared to the use of FOB. The newer video stylets are equipped with a high-resolution camera with a wide field of view (in some cases reaching 60°) and some with an anti-fog coating. The stylet can be inserted in most difficult airways and the malleable rod can be removed in case extra flexibility is needed, such as during nasotracheal intubation or intubating through a laryngeal mask. The range of movements of video stylets has been improved and in some cases is greater than 90°. Hence, flexible video stylets may be a promising option and could be preferred over the CLBI by anesthesiologists not routinely using the FOB. Nonetheless, the performance of the newer flexible video stylets have not yet been extensively compared with DL, VLS and CLBI.

We conducted a single-center, observational, prospective, cross-sectional study testing the performance of a flexible video stylet in the setting of simulated normal airways in a population of attendings/consultants with experience in several ETI devices but without experience with this device. We hypothesized that the flexible video stylet would have had lower performance compared to DL and VLS but superior to the CLBI approach.

## 2. Materials and Methods

We conducted an observational, descriptive, cross-sectional manikin trial at our simulation center (“Cristian Ilardi” Simulation Center) at the University of Catania.

### 2.1. Study Participants

The simulation study involved consultants in Anesthesia, Intensive Care and Pain Therapy, all of them working in a single trust (Policlinico “G.Rodolico—San Marco” Hospital of Catania). We recorded age, gender, year of completion of training and their clinical knowledge with each of the five airway techniques tested for adult ETI.

### 2.2. Study Development

Our study evaluated five approaches to ETI in a simulated normal airway scenario. Our simulation center is equipped with the Larry Intubation Trainer (Armstrong Medical Inc., 575 Knightsbridge Parkway, P.O. Box 700, Lincolnshire, IL 60069-0700, USA) manikin, which was used for this study. The Larry Intubation Trainer was positioned on a rigid board in a lighted room; the height of the board was placed at the level of the xiphoid process of each participant but it was changed at their discretion. Four independent operators (SM, MS, GS, FL) carried out the study, providing standardized initial teaching sessions, chronometer assessment and recording scores and times. A standardized 10 min teaching session was provided to participants on study methods and devices. Participants were not permitted to look at someone else’s attempts, in order to exclude any learning influence.

Five techniques/devices were studied with randomized sequence for device order (sealed envelopes):

A. DL, using a Macintosh laryngoscope blade size 3 (Mercury Medical, Clearwater, FL, USA);
B. VLS with screen on device, with the McGrath MAC blade X3 (McGrath; Aircraft Medical Ltd., Edinburgh, UK);
C. VLS with distant monitor, with Glidescope (Glidescope Verathon Inc., 20001 North Creek Parkway, Bothell, WA 98011, USA);
D. Articulating flexible video stylet, the ProVu^®^ Video stylet (Flexicare Inc., Irvine, CA, USA) introduced with the aid of Macintosh DL to open the upper airways.
E. CLBI approach, with the Macintosh DL and the use of a disposable bronchoscope (aScope™ 4 Broncho Regular endoscope, Ambu A/S Baltorpbakken 13, DK-2750 Ballerup, Denmark).

This study received no financial support. All the airway devices were already available at our Simulation Center where we conduct training of residents, apart from the video stylet ProVu that was provided for free by a local company that had no role and no influence in the whole study (design, methodology, setting, data and results, writing and editing of the manuscript). The study procedures were performed using a lubricated ETT (7.5 mm internal diameter was the default one). In the case of the two VLS approaches, a semi-rigid stylet was preventively placed into the ETT (hockey stick-shaped). However, upon request by each participant, a stylet was available during DL attempts. We took care of periodically wetting the manikin and the ETT with a lubricant. As in other studies performed by our group, we chose two primary outcomes: the first and more obvious one was the success rate (SR) in achieving ETI, whilst the second one was the corrected time to intubation (cTTI).

Concerning the SR, the study design allowed up to three ETI attempts for each approach for each study participant. The success of the procedure was declared after positive lung insufflations confirmed by the research team. Conversely, failure was declared for each attempt lasting over 1 min, or if procedure resulted in esophageal intubation.

The cTTI was the time taken to intubate but weighted for the number of attempts. The research team activated the chronometer the moment the operator grasped the airway device and the time count was stopped as soon as the participant declared that ETT passed vocal cords. In order to obtain the cTTI, 1 min was added for each previously failed attempt to the timing registered on the chronometer. As an example, an ETT successfully performed after 15 s after a previously failed attempt resulted in a cTTI of 75 s (15 + 1 min for one previous failure). If the participant failed all the tries, we assigned an overall count of 3 min (180 s).

Our secondary endpoints included uncorrected time to intubation (uTTI), corrected time to ventilation (cTTV, with chronometer stopped at successful bag inflation) and ratings on the ease of intubation procedure using a Likert scale (from 1 to 10, with 10 meaning very easy). Other variables recorded were the clinical experience of each operator evaluated through the estimated number of previous ETTs performed with each study device, the glottic visualization described according to the Cormack–Lehane classification [30] and the Percentage Of Glottis Opening (POGO) scale, which varied from 0% to 100% [31]. Although such scales are used and validated for DL and VLS, participants were asked to estimate the POGO and Cormack–Lehane for their attempt also with CLBI and ProVu.

### 2.3. Statistical Analysis

We explored the data distribution with Shapiro–Wilk test and reported data as numbers (percentages) in case of categorical variables and analyses were conducted using the Fisher’s exact test. Conversely, continuous variables are described according to their distribution, hence mean and standard deviation for normally distributed data, or as median and interquartile range [IQR] in case of not normal distribution. Accordingly, we used the appropriate paired test (*t*-Student test or Wilcoxon’s rank test). Differences between groups in terms of categorical or continuous variables were considered significant if *p* value was below 0.05.

## 3. Results

We recruited 42 volunteers for this study (May–June 2023). The participants had a mean age of 46.5 ± 8.4 years and 47.6% were males (*n* = 20). The mean number of years after completion of their training in anesthesia and intensive care was 15 ± 9. We report for each device the performance in terms of the SR, cTTI and uTTI (Table 1).

The comparison of the performance of the five devices in terms of the SR (at first and third attempt), and their different cTTIs are shown in Table 2.

We found several differences for the SR analysis. By the first attempt, the DL and the VLSs had no differences between each other, but performed significantly better than the ProVu and the CLBI; in turn, the ProVu performed significantly better than the CLBI (*p* = 0.016). However, by the third attempt, there were no significant differences between the three laryngoscopes and the ProVu. The only significant differences in the SR by the last attempt were the superiority of the three laryngoscopes and of the ProVu compared to the CLBI.

In terms of the cTTI, all the comparisons yielded significant differences apart from the analysis comparing the two VLSs (*p* = 0.775). The DL was the best-performing device followed sequentially by the two VLSs, the ProVu and the CLBI approach.

Table 3 reports the self-judged experience of the participants with each device/technique and shows the results of the ease of use, the POGO and the Cormack–Lehane evaluations for all the techniques.

The participants had significantly higher reported experience with the DL compared to the other four techniques (all *p* < 0.001), and no differences in experience between the two VLSs (*p* = 0.28). They reported significantly higher expertise in both VLSs compared to the CLBI and the ProVu (all *p* < 0.001), and more experience in the CLBI compared to the ProVu (*p* < 0.001). This pattern and magnitude of statistical differences were almost identical to the reported ease of use, with the only exception of no differences reported in the ease of use between the CLBI and the ProVu (*p* = 0.48). The two VLSs had higher POGO results compared to the ProVu, with no other differences noted between the devices/techniques. A good visualization of the vocal cords with Cormack–Lehane grade 1 or 2a was achieved by 40 (DL), 41 (Glidescope), 42 (McGrath), 36, (CLBI) and 34 (ProVu) participants.

### Sensitivity Analyses

The analyses conducted on the uTTI (the time recorded for the ETI success not corrected for previous failures) mostly confirmed the primary analysis obtained for the cTTI. The only change was that the uTTI became not significantly different between the CLBI and the ProVu (*p* = 0.54, whilst for cTTI, was *p* = 0.004). The analyses on the cTTV and uTTV showed parallel results to the ones conducted on cTTI and uTTI, respectively (results available on request).

## 4. Discussion

We conducted a single-center, observational, prospective, cross-sectional study testing the performance of a flexible video stylet in the setting of simulated normal airways in a population of consultants with no prior clinical experience with this device. We hypothesized that the flexible video stylet would have a lower performance compared to the DL and VLS (a longer cTTI but comparable SR by the third attempt), and a superior performance compared to the CLBI approach. Of course, our results should be interpreted in light of the experience with each of the devices, and in the context of a normal airway simulated scenario.

With this background, we found that the ProVu had a significantly worse performance in the DL and VLSs in terms of the cTTI and of SR at the first attempt, a result somewhat expected and fully justifiable. However, by the third attempt, we found no differences in the SR between the ProVu and the three laryngoscope techniques, suggesting that, after minimal exposure to the device, the operators were truly able to achieve some performance. This reduced the gap with other devices (DL and VLS) for which they have been trained and that they use frequently. Moreover, despite the significantly higher experience reported for the CLBI, the ProVu performed significantly better both in terms of the SRs and in the cTTI and uTTI. However, it must be noted that the reported experience with the CLBI approach in our study population was still suboptimal (median of 2 out of 10). Therefore, it is possible that our results could be different in participants who have mastered the use of FOB. We think the superiority of the ProVu is related to its user-friendly design, being of a comparable size to a laryngoscope and being similarly handled with one hand only. Conversely, the FOB requires both hands for manipulation, it is much bigger than a laryngoscope and has a challenging learning curve. Hence, our results may be encouraging for the use of flexible articulating video stylets. Interestingly, in terms of the visualization of the vocal cords (i.e., Cormack–Lehane grade 1 and 2a), the results of the ProVu and CLBI were not dissimilar from the three laryngoscopes.

Interestingly, a case series of three “awake-intubations” performed with the ProVu under propofol and remifentanil has reported a 100% SR at first attempt in patients with predicted difficult airways (limited mouth opening secondary to radiotherapy; previous exenteration, hemi-maxillectomy and scapular free flap formation and cervical fixation) [32]. In a small simulation study on 16 participants, Nikolla et al. found that the articulating video stylet may perform similarly to the traditional rigid stylet when used with Glidescope VLS, although the rigid stylet achieved ETI significantly faster, by over 3 s [33]. However, once again, the experience with the device seems crucial, as in this study, over 80% of the participants had experience with a rigid stylet and no one had used the ProVu.

Regarding the other results of our study, it is not entirely surprising to find that the DL and VLSs had the best performance, with the participants reporting good to excellent experience with such devices. Whilst the SR in our study was not different between the DL and the two VLSs, the DL had a significantly faster performance (cTTI and uTTI), despite a very similar Cormack–Lehane grade. This result could be explained by the easier task of directing the ETT through the vocal cords when performing the DL; indeed, VLS produces better visualization but at the expense of the visualization axis, and for this reason, once again, the main challenge highlighted with VLS is to properly guide the ETT to the target. However, recent evidence supports the usefulness of VLS as a first device for the management of airways in emergency departments, although in this multicenter randomized trial, the vast majority of the participants were at an early stage of their career (~95% in training), with no anesthesiology background (~95%), and had significant experience in VLS (~95%) [14]. Notably, the advantages of VLS disappeared in the operators with at least 100 ETI procedures, suggesting again the importance of training in ETI and confidence with the device used. Considering the challenge of directing the ETT through the vocal cords with VLS, the CLBI seemed a promising method as it combines a better visualization with the fine manipulation of the ETT. Indeed, from a conceptual standpoint, it seems intuitive to combine the advantages of airway visualization offered by DL/VLS with the precision of guiding the ETT using FOB. The CLBI has been implemented in several clinical reports [34,35,36]. However, mastering the use of FOB is not always common among anesthesiologists, and for such reason, the so-called CLBI has produced variable results, with more encouraging findings in studies investigating CLBI at the consultant level and worse when the performance was evaluated in a resident population [26,27,28,29]. However, it should be considered that both the ProVu and CLBI approach might be valuable for the education of younger trainees, paramedics and novices in general, as they may allow for optimal supervision during the intubation maneuver. Indeed, both the mentor and resident look at the same screen and share an identical view. It has already been shown that novices may learn more easily from supervisors using the CLBI method [37]. Conversely, it is more challenging to teach laryngoscopy as the teacher and trainee do not have a simultaneous and identical view.

*Strengths and Limitations*

Some strengths and several limitations of our study deserve to be acknowledged. A strength of our investigation is that it represents the largest study assessing the performance of the ProVu, a novel articulating video stylet. Moreover, our study achieved a relatively large sample size when compared to other simulation studies reported in the literature and involving anesthesiology consultants.

We believe that a second strength is the statistical approach using a parameter (cTTI) accounting for previously failed attempts. Indeed, from a clinical standpoint, each failed attempt means a potentially life-threatening risk with desaturation and/or bleeding and/or increase in airway secretions. Such decision considers, for instance, a hypothetical device with a short TTI but low SR, which may be seen as outperforming other devices with a longer TTI but greater SR. Whether this decision is agreed upon or not, we have been fully clear in this approach, and we have already used it in previous simulation studies conducted and published, while we have found several studies that have been vague in terms of handling the timing for failed attempts. Nonetheless, we also reported the findings of the uTTI, which were absolutely comparable to those obtained for the cTTI. Finally, a third strength is the approach with randomization in the order of performing the ETI with each technique.

There are several limitations in our study. First, it is a single-center study requiring external validation. Second, the results are confined to a population of consultants with no experience in the ProVu and little experience in CLBI. Indeed, whilst the population may seem homogeneous (anesthesia consultants), their experience in airway management was highly variable, as was the experience with some devices. In particular, the experience in CLBI was highly variable, with an interquartile range much larger than with the other devices.

Finally, the role of simulation in the field of airways is of the utmost importance. For instance, a systematic review and meta-analysis [38] suggests an important role of simulation to train healthcare professionals in the advanced management of the airway. However, all simulation studies, including ours, suffer from several intrinsic issues. Of course, simulation scenarios are not designed to reproduce real-life challenges encountered commonly and affecting the performance of each technique. For instance, the presence of secretions and bleeding (among others) cannot be reproduced, nor can be replicated the emotional stress of the operator (i.e., anxiety due to worsening the clinical situation, such as the occurrence of desaturation) [39]. Moreover, several studies in both adult and pediatric settings have questioned the similarity between manikins and the real human anatomy [40,41].

## 5. Conclusions

In a simulation study conducted in normal airway circumstances with consultants with experience in endotracheal intubation but having no prior experience with ProVu, we showed encouraging results for this articulating video stylet technique. In particular, although the participants required a longer time to perform intubation (corrected by the number of attempts), the difference in the success rate disappeared by the third attempt. Hence, despite the participants being novice to the use of this technique, the ProVu success rate after three attempts was similar to the ones obtained with direct laryngoscopy and videolaryngoscopy. Moreover, the ProVu showed superiority compared to the combined laryngo-bronchoscopy approach, which may be perceived as complex by personnel not routinely exposed to the use of bronchoscopy. Further studies are warranted to replicate and validate our findings.

Take-home message: In summary, we showed that, despite no prior experience with an articulating video stylet, the consultants reached a reasonable performance with the ProVu compared to the techniques in which they are much more experienced, such as direct and video laryngoscopy. Of note, the difference in the success rate between the ProVu and direct and video laryngoscopes disappeared by the third attempt. Readers must be aware that these results were obtained in a simulation study conducted in normal airway circumstances, and the results may be different in difficult airway scenarios, or under real conditions, or in personnel with prior experience with articulating video stylets. It is advisable to conduct further studies to provide external validation to our findings.

## Figures and Tables

**Table 1:** Success rate (SR), corrected and uncorrected time to intubation (cTTI and uTTI, respectively) for each of the five techniques studied. The statistical differences in performance between techniques are provided separately in Table 2. DL: direct laryngoscopy; CLBI: combined laryngo-bronchoscope intubation.

	DL	Glidescope	McGrath	CLBI	ProVu
SR at 1st attempt	41/42 (97%)	37/42 (88%)	36/42 (86%)	16/42 (38%)	28/42 (67%)
SR at 2nd attempt	0	2/42 (5%)	5/42 (12%)	6/42 (14%)	6/42 (14%)
SR at 3rd attempt	0	1/42 (2%)	0	11/42 (26%)	3/42 (7%)
Failure by 3rd attempt	1/42 (3%)	2/42 (5%)	1/42 (2%)	9/42 (22%)	5/42 (12%)
cTTI	14.8 [13.0–19.0]	22.9 [16.8–34.9]	21.5 [14.4–45.9]	96.3 [46.6–174.7]	44.7 [34.0–100.1]
uTTI	14.7 [13.0–18.8]	22.1 [16.3–29.3]	18.7 [14.3–35.3]	38.5 [31.1–51.4]	37.1 [29.7–46.2]

**Table 2:** Differences in success rate (SR), corrected and uncorrected time to intubation (cTTI and uTTI, respectively) between techniques are shown. DL: direct laryngoscopy; CLBI: combined laryngo-bronchoscope intubation. In bold are the statistically significant results.

cTTI	DL	Glidescope	McGrath	CLBI
Glidescope	**0.033**			
McGrath	**0.033**	0.775		
CLBI	**<0.001**	**<0.001**	**<0.001**	
ProVu	**<0.001**	**0.002**	**<0.001**	**0.004**
**SR at 1st attempt**				
Glidescope	0.202			
McGrath	0.109	1.0		
CLBI	**<0.001**	**<0.001**	**<0.001**	
ProVu	**<0.001**	**0.035**	**0.071**	**0.016**
**SR at 3rd attempt**				
Glidescope	1.0			
McGrath	1.0	1.0		
CLBI	**<0.001**	**0.003**	**<0.001**	
ProVu	0.241	0.616	0.241	**0.038**

**Table 3:** Experience with technique and other secondary outcomes (ease of use, Cormack–Lehane and percentage of glottis opening [POGO]). The ease of use for each technique is reported on a Likert-scale (1–10). Results are reported in median and interquartile range. DL: direct laryngoscopy; CLBI: combined laryngo-bronchoscope intubation.

Device (Participants, *n* = 42)	Experience with Technique (1–10)	Ease of Use (1–10)	POGO (%)	Cormack–Lehane (1, 2a, 2b, 3, 4)
DL	10 (0)	10 (0)	100 (25)	28-12-0-1-1
Glidescope	8 (5)	9 (2)	100 (0)	32-9-1-0-0
McGrath	7 (6)	10 (2)	100 (25)	30-12-0-0-0
CLBI	2 (6)	6 (3)	100 (21)	31-5-1-2-3
ProVu	0 (0)	6 (5)	100 (25)	28-6-2-1-5

## Data Availability

Data available on reasonable request to the corresponding author.

## References

[1] Santou N., Ueta H., Nakagawa K., Hata K., Kusunoki S., Sadamori T., Takyu H., Tanaka H. (2023). A comparative study of Video laryngoscope vs Macintosh laryngoscope for prehospital tracheal intubation in Hiroshima, Japan. Resusc. Plus.

[2] Liti A., Giusti G.D., Gili A., Giontella M., Dell’Omo S., Camerlingo V., Fronteddu A., Galazzi A., Bambi S. (2020). Insertion of four different types of supraglottic airway devices by emergency nurses. A mannequin-based simulation study. Acta Biomed..

[3] Nielsen R.P., Nikolajsen L., Paltved C., Aagaard R. (2021). Effect of simulation-based team training in airway management: A systematic review. Anaesthesia.

[4] Wong H.Y., Johnstone C., Dua G. (2021). Developing a simulation programme to train airway management during the COVID-19 pandemic in a tertiary-level hospital. BMJ Simul. Technol. Enhanc. Learn..

[5] Rothkrug A., Mahboobi S.K. (2022). Simulation Training and Skill Assessment in Anesthesiology. StatPearls.

[6] Sanfilippo F., Tigano S., La Rosa V., Morgana A., Murabito P., Oliveri F., Longhini F., Astuto M. (2022). Tracheal intubation while wearing personal protective equipment in simulation studies: A systematic review and meta-analysis with trial-sequential analysis. Braz. J. Anesthesiol..

[7] Mulcaster J.T., Mills J., Hung O.R., MacQuarrie K., Law J.A., Pytka S., Imrie D., Field C. (2003). Laryngoscopic intubation: Learning and performance. Anesthesiology.

[8] Wang H.E., Seitz S.R., Hostler D., Yealy D.M. (2005). Defining the learning curve for paramedic student endotracheal intubation. Prehospital Emerg. Care.

[9] Malik M.A., O’Donoghue C., Carney J., Maharaj C.H., Harte B.H., Laffey J.G. (2009). Comparison of the Glidescope, the Pentax AWS, and the Truview EVO2 with the Macintosh laryngoscope in experienced anaesthetists: A manikin study. Br. J. Anaesth..

[10] Saul S.A., Ward P.A., McNarry A.F. (2023). Airway Management: The Current Role of Videolaryngoscopy. J. Pers. Med..

[11] Hansel J., Rogers A.M., Lewis S.R., Cook T.M., Smith A.F. (2022). Videolaryngoscopy versus direct laryngoscopy for adults undergoing tracheal intubation: A Cochrane systematic review and meta-analysis update. Br. J. Anaesth..

[12] Cox L., Tebbett A. (2022). Videolaryngoscopy versus direct laryngoscopy for endotracheal intubation of cardiac arrest patients in hospital: A systematic literature review. Resusc. Plus.

[13] Ho C.H., Chen L.C., Hsu W.H., Lin T.Y., Lee M., Lu C.W. (2022). A Comparison of McGrath Videolaryngoscope versus Macintosh Laryngoscope for Nasotracheal Intubation: A Systematic Review and Meta-Analysis. J. Clin. Med..

[14] Prekker M.E., Driver B.E., Trent S.A., Resnick-Ault D., Seitz K.P., Russell D.W., Gaillard J.P., Latimer A.J., Ghamande S.A., Gibbs K.W. (2023). Video versus Direct Laryngoscopy for Tracheal Intubation of Critically Ill Adults. N. Engl. J. Med..

[15] Ruetzler K., Imach S., Weiss M., Haas T., Schmidt A.R. (2015). Comparison of five video laryngoscopes and conventional direct laryngoscopy: Investigations on simple and simulated difficult airways on the intubation trainer. Der Anaesthesist.

[16] Malik M.A., Maharaj C.H., Harte B.H., Laffey J.G. (2008). Comparison of Macintosh, Truview EVO2, Glidescope, and Airwayscope laryngoscope use in patients with cervical spine immobilization. Br. J. Anaesth..

[17] Savoldelli G.L., Schiffer E., Abegg C., Baeriswyl V., Clergue F., Waeber J.L. (2009). Learning curves of the Glidescope, the McGrath and the Airtraq laryngoscopes: A manikin study. Eur. J. Anaesthesiol..

[18] Maharaj C.H., McDonnell J.G., Harte B.H., Laffey J.G. (2007). A comparison of direct and indirect laryngoscopes and the ILMA in novice users: A manikin study. Anaesthesia.

[19] Sanfilippo F., Chiaramonte G., Sgalambro F. (2017). Video Laryngoscopes and Best Rescue Strategy for Unexpected Difficult Airways: Do Not Forget a Combined Approach with Flexible Bronchoscopy!. Anesthesiology.

[20] Sgalambro F., Sanfilippo F., Santonocito C., Caltavuturo C., Grillo C. (2014). Virtual laryngoscopy and combined laryngoscopic-bronchoscopic approach for safe management of obstructive upper airways lesions. Br. J. Anaesth..

[21] Nedrud S.M., Baasch D.G., Cabral J.D., McEwen D.S., Dasika J. (2021). Combined Video Laryngoscope and Fiberoptic Nasal Intubation. Cureus.

[22] Yukihiro I., Komasawa N., Fujiwara S., Tsuji E., Minami T. (2016). Efficacy of a Bronchofiberscope in Combination with the GlideScopee in a Difficult Airway Patient. SAGE Open Med. Case Rep..

[23] Stein M.L., Park R.S., Kiss E.E., Adams H.D., Burjek N.E., Peyton J., Szmuk P., Staffa S.J., Fiadjoe J.E., Kovatsis P.G. (2023). Efficacy of a hybrid technique of simultaneous videolaryngoscopy with flexible bronchoscopy in children with difficult direct laryngoscopy in the Pediatric Difficult Intubation Registry. Anaesthesia.

[24] Lim W.Y., Wong P. (2019). Awake supraglottic airway guided flexible bronchoscopic intubation in patients with anticipated difficult airways: A case series and narrative review. Korean J. Anesthesiol..

[25] Swain A., Bhagat H., Gupta V., Salunke P., Panda N.B., Sahu S. (2020). Intubating Laryngeal Mask Airway-assisted Flexible Bronchoscopic Intubation Is Associated with Reduced Cervical Spine Motion When Compared With C-MAC Video Laryngoscopy-guided Intubation: A Prospective Randomized Cross Over Trial. J. Neurosurg. Anesth..

[26] Nishikawa K., Hukuoka E., Kawagishi T., Shimodate Y., Yamakage M. (2011). Efficacy of the Airtraq((R)) laryngoscope with a fiberoptic bronchoscope compared with that of Airtraq((R)) alone for tracheal intubation: A manikin study. J. Anesth..

[27] Sanfilippo F., Sgalambro F., Chiaramonte G., Santonocito C., Burgio G., Arcadipane A. (2019). Use of a Combined Laryngo-Bronchoscopy Approach in Difficult Airways Management: A Pilot Simulation Study. Turk. J. Anaesthesiol. Reanim..

[28] La Via L., Merola F., Messina S., Sanfilippo G., Tornitore F., Lombardo F., Sanfilippo M., Tigano S., Sanfilippo F. (2023). Use of combined laryngo-bronchoscopy intubation approach in a simulated difficult airway scenario with cervical stabilization. Signa Vitae.

[29] La Via L., Messina S., Merola F., Tornitore F., Sanfilippo G., Santonocito C., Noto A., Longhini F., Sanfilippo F., Astuto M. (2023). Combined laryngo-bronchoscopy intubation approach in the normal airway scenario: A simulation study on anesthesiology residents. Signa Vitae.

[30] Krage R., van Rijn C., van Groeningen D., Loer S.A., Schwarte L.A., Schober P. (2010). Cormack-Lehane classification revisited. Br. J. Anaesth..

[31] Levitan R.M., Ochroch E.A., Kush S., Shofer F.S., Hollander J.E. (1998). Assessment of airway visualization: Validation of the percentage of glottic opening (POGO) scale. Acad. Emerg. Med. Off. J. Soc. Acad. Emerg. Med..

[32] Harrison S.L., Ahmad I., Elwen F., Curtis A., Dua G., Surda P., Johnstone C. (2021). Awake tracheal intubation with the ProVu™ video stylet: A case series. Anaesth. Rep..

[33] Nikolla D.A., Boulet S., Carlson J.N. (2023). Comparison of Rigid and Articulating Video Stylets During Simulated Endotracheal Intubation with Hyperangulated Video Laryngoscopy. J. Emerg. Med..

[34] Gomez-Rios M.A., Nieto Serradilla L. (2011). Combined use of an Airtraq(R) optical laryngoscope, Airtraq video camera, Airtraq wireless monitor, and a fibreoptic bronchoscope after failed tracheal intubation. Can. J. Anaesth..

[35] Greib N., Stojeba N., Dow W.A., Henderson J., Diemunsch P.A. (2007). A combined rigid videolaryngoscopy-flexible fibrescopy intubation technique under general anesthesia. Can. J. Anaesth..

[36] Sharma D., Kim L.J., Ghodke B. (2010). Successful airway management with combined use of Glidescope videolaryngoscope and fiberoptic bronchoscope in a patient with Cowden syndrome. Anesthesiology.

[37] Hung W.T., Yang M.W., Lin C.Y. (2001). Evaluation of learning effectiveness in endotracheal intubation by the use of a laryngoscope in combination with a flexible fiberoptic bronchoscope. Acta Anaesthesiol. Sin..

[38] Kennedy C.C., Cannon E.K., Warner D.O., Cook D.A. (2014). Advanced airway management simulation training in medical education: A systematic review and meta-analysis. Crit. Care Med..

[39] Doleman B., Blackwell J., Karangizi A., Butt W., Bhalla A., Lund J.N., Williams J.P. (2016). Anaesthetists stress is induced by patient ASA grade and may impair non-technical skills during intubation. Acta Anaesthesiol. Scand..

[40] Schebesta K., Hupfl M., Rossler B., Ringl H., Muller M.P., Kimberger O. (2012). Degrees of reality: Airway anatomy of high-fidelity human patient simulators and airway trainers. Anesthesiology.

[41] Schebesta K., Hupfl M., Ringl H., Machata A.M., Chiari A., Kimberger O. (2011). A comparison of paediatric airway anatomy with the SimBaby high-fidelity patient simulator. Resuscitation.

